# *Serine carboxypeptidase 46* Regulates Grain Filling and Seed Germination in Rice (*Oryza sativa L*.)

**DOI:** 10.1371/journal.pone.0159737

**Published:** 2016-07-22

**Authors:** Zhiyong Li, Liqun Tang, Jiehua Qiu, Wen Zhang, Yifeng Wang, Xiaohong Tong, Xiangjin Wei, Yuxuan Hou, Jian Zhang

**Affiliations:** 1 State Key Lab of Rice Biology, China National Rice Research Institute, Hangzhou, 311400, P.R. China; 2 China National Rice Research Institute, Hangzhou, 311400, P.R. China; Institute of Genetics and Developmental Biology, Chinese Academy of Sciences, CHINA

## Abstract

Serine carboxypeptidase (*SCP*) is one of the largest groups of enzymes catalyzing proteolysis for functional protein maturation. To date, little is known about the function of *SCPs* in rice. In this study, we present a comprehensive analysis of the gene structure and expression profile of 59 rice *SCPs*. *SCP46* is dominantly expressed in developing seeds, particularly in embryo, endosperm and aleurone layers, and could be induced by ABA. Functional characterization revealed that knock-down of *SCP46* resulted in smaller grain size and enhanced seed germination. Furthermore, *scp46* seed germination became less sensitive to the ABA inhibition than the Wild-type did; suggesting *SCP46* is involved in ABA signaling. As indicated by RNA-seq and qRT-PCR analysis, numerous grain filling and seed dormancy related genes, such as *SP*, *VP1* and *AGPs* were down-regulated in *scp46*. Yeast-two-hybrid assay also showed that SCP46 interacts with another ABA-inducible protein DI19-1. Taken together, we suggested that *SCP46* is a master regulator of grain filling and seed germination, possibly *via* participating in the ABA signaling. The results of this study shed novel light into the roles of SCPs in rice.

## Introduction

Proteolysis catalyzed by peptidase is an important post-translational modification for functional protein maturation. Based on the location of the cleavage site within the substrate, peptidases are divided into endopeptidase and exopeptidase. Endopeptidase splits internal peptide bonds within the protein, whereas exopeptidase can only detach the terminal amino acids of the protein chain. According to the terminus position of the peptide studied, an exopeptidase can be further classified into either an aminopeptidase or carboxypeptidase. Aminopeptidases usually cleave one or two residues each time from the N-terminus. On the contrary, carboxypeptidases cleave peptides from the C-terminus at a speed of one residue each time. Serine carboxypeptidase (SCP) is a type of acid carboxypeptidase whose optimum hydrolysis activity is achieved under acidic pH range. Hayashi et al (1973) named this group of enzymes as “serine carboxypeptidase” because they could be inhibited by a specific serine protease inhibitor DFP [1]. In protein structure, SCPs have a conserved Ser-Asp-His catalytic triad. Though these residues are dispersed in the primary amino acid sequence, the triad residues are physically aggregated in the protein tertiary structure to create a nucleophilic serine for substrate hydrolysis [2]. SCPs also have another conserved oxyanion hole motif, whose function is to stabilize the enzyme-substrate interaction during the substrate protein hydrolysis. Given the special enzymatic features in protein hydrolysis, SCPs have been widely used as molecular biology tool enzymes for determination, exchanging and synthesis of the C-terminal sequence in peptides [3].

Recently, increasing evidences have shown that SCPs play crucial roles in protein transport, targeting and processing. In humans, defects in SCP are associated with severe diseases ranging from obesity to epilepsy to neurodegeneration [4,5]. Besides humans, *SCPs* and *SCP-like (SCPL)* genes are widely distributed in animals, bacterium, fungi as well as higher plants [6–13]. A genome survey conducted by Tripathi et al (2006) revealed 54 and 66 *SCPL* genes in Arabidopsis and rice respectively [11]. By using the same hidden Markov models but different criteria, Feng et al (2009) identified 54 and 71 *SCPL* genes in Arabidopsis and rice. A large number of the Arabidopsis *SCPLs* showed rich alternative splicing models, while this phenomenon barely occurred in rice *SCPLs* [2]. The Arabidopsis *SCP* members displayed divergent spatial-expression patterns though they were phylogenetically closely related, suggesting versatile roles of *SCPs* in plant development [7]. Indeed, when *ECS1 (Extra Carpels and Seeds)*, encoding a serine carboxypeptidase, was ectopically expressed in Arabidopsis, the carpel and silique seed number were significantly increased. Genetic analysis of *ecs1* and *bri1-5* (*BRassinosteroid Insensitive 1–5*) implied an important role of ECS1 in BR (brassinosteroid) signaling [14]. Additionally, another Arabidopsis SCP protein BRS1 was found to be involved in early event of BR signaling [15]. In germinating wheat grains, *carboxypeptidase III (CPIII)* is highly accumulated in the scutellum at 2–3 days after imbibition, indicating a role of *CPIII* in mobilization of starchy-endosperm proteins. Moreover, in situ nuclear DNA fragmentation was detected in cells with high *CPIII* expression, thus the authors proposed that *CPIII* may also be involved in programmed cell death during the development of the vascular tissue in wheat [16]. Unlike its wheat ortholog *CPIII*, *PsCP* from pea (*Pisum sativum L*.) does not participate in the mobilization of storage nutrients, but a predominant expression pattern in seed and selective hormone-induced, in particular GA-induced, expression pattern in seedlings still hinted of a very important function of *PsCP* in reproductive and vegetative developing tissues in dicotyledoneous plants [17]. Bienert et al (2012) isolated two extracellular serine carboxypeptidase III genes from tobacco (*Nicotiana tabacum*), *NtSCP1* and *NtSCP2*. Both of these genes displayed a constitutive expression among all the checked tissues, but only purified His-tagged NtSCP1 had carboxypeptidase activity *in vitro*. Functional characterization revealed that *NtSCP1* regulates cell elongation in flower and hypocotyls. Unfortunately, this study did not further explore the relationship between *NtSCP1* and phytohormone GA, as there were already some cases showing SCPs like *CPIII* and *PsCP* are highly associated with GA controlled cell elongation [18]. In addition to the peptidase function, the emerging roles of SCPLs as acyltransferases were gradually recognized [12,19,20]. SCPL enzymes were shown to catalyze the region specific formation of diacylglucose (1,2-di-O-acylglucose) using the glucose esters 1-O-acylglucose as acyl donors [21]. So far, several publications have reported that SCPL acyltransferases are involved in secondary metabolism, herbicide detoxication, development, biotic and abiotic stress responses in a broad range of plant species, which greatly enhanced understanding of SCPs and SCPLs [7,12,22–26].

Rice (*Oryza sativa* L.) is one of the major crops in the world, feeding over half of the global population. Besides its economical importance, rice is also an ideal model plant for gene functional studies due to its released genome sequence, ample genetic resources, mature transformation system and co-linearity with other grasses [27]. In the past few decades, over 2000 rice genes have been cloned and functionally characterized, representing a tremendous progress in plant molecular biology research (http://www.ricedata.cn/gene/) [28]. Regarding the *SCPs* in rice, despite scientific exploration of the spatial- and hormone-induced expression pattern of the family members in the 1990s, the knowledge on rice *SCPs* remains rather poor with only a few exceptions over the last 20 years [29]. For example, *OsBISCPL1* encoding a putative *SCPL* was found to positively regulate rice resistance against bacterial pathogens by elevating defense genes such as *PR1*, *PR2*, *PR5* and *PDF1*.*2*. There was evidence showing that *OsBISCPL1* may be related to the oxidative stress responses as well [30]. Li et al (2011) cloned another rice *SCP* gene *GS5* which is a major QTL site determining rice grain size. [31]. In this study, we systematically analyzed the gene structure, tissue expression profile of rice *SCP* family. Employing a reverse genetic strategy, we revealed that *SCP46*, a seed-dominantly expressed gene, controls rice grain filling and seed germination by involvement in the ABA signaling. The results of this research shed novel light into the function of *SCPs* in rice.

## Results

### Rice *SCP* genes exhibit diverse expression patterns

**Fig 1:**
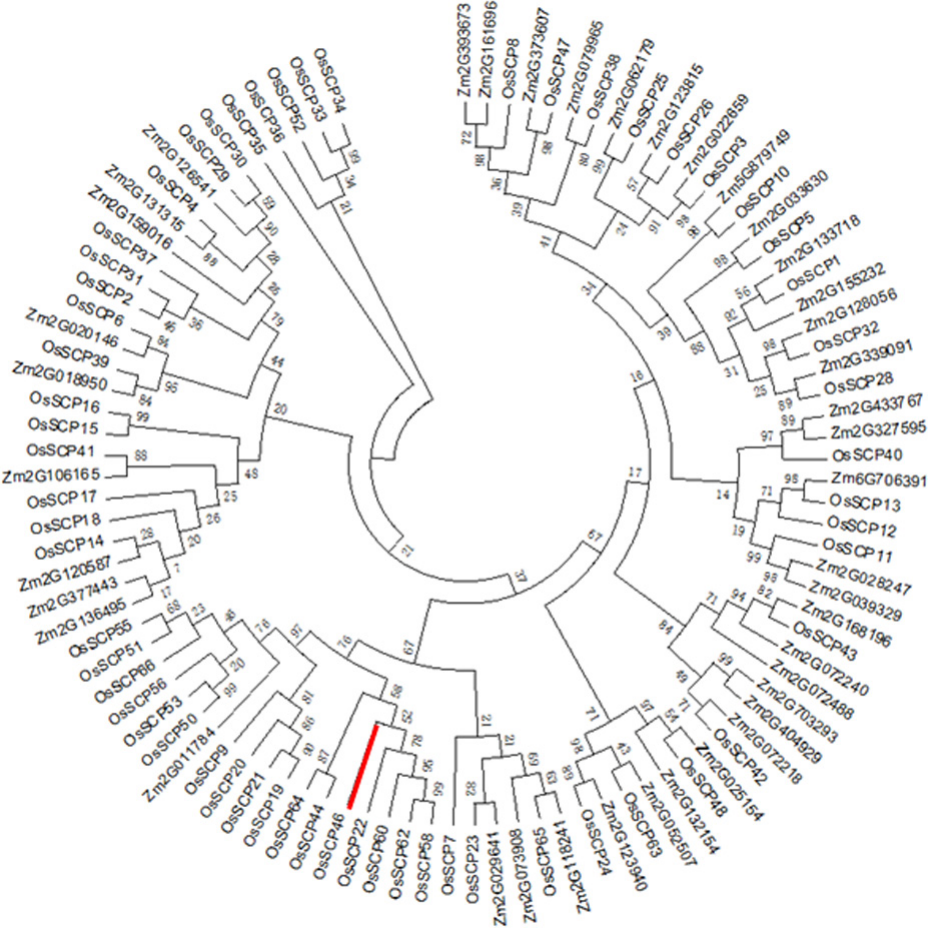
Phylogenetic analysis of rice and maize SCPs. The branch length scale bar indicates the evolutionary distance. Red line corresponds to SCP46.

**Fig 2:**
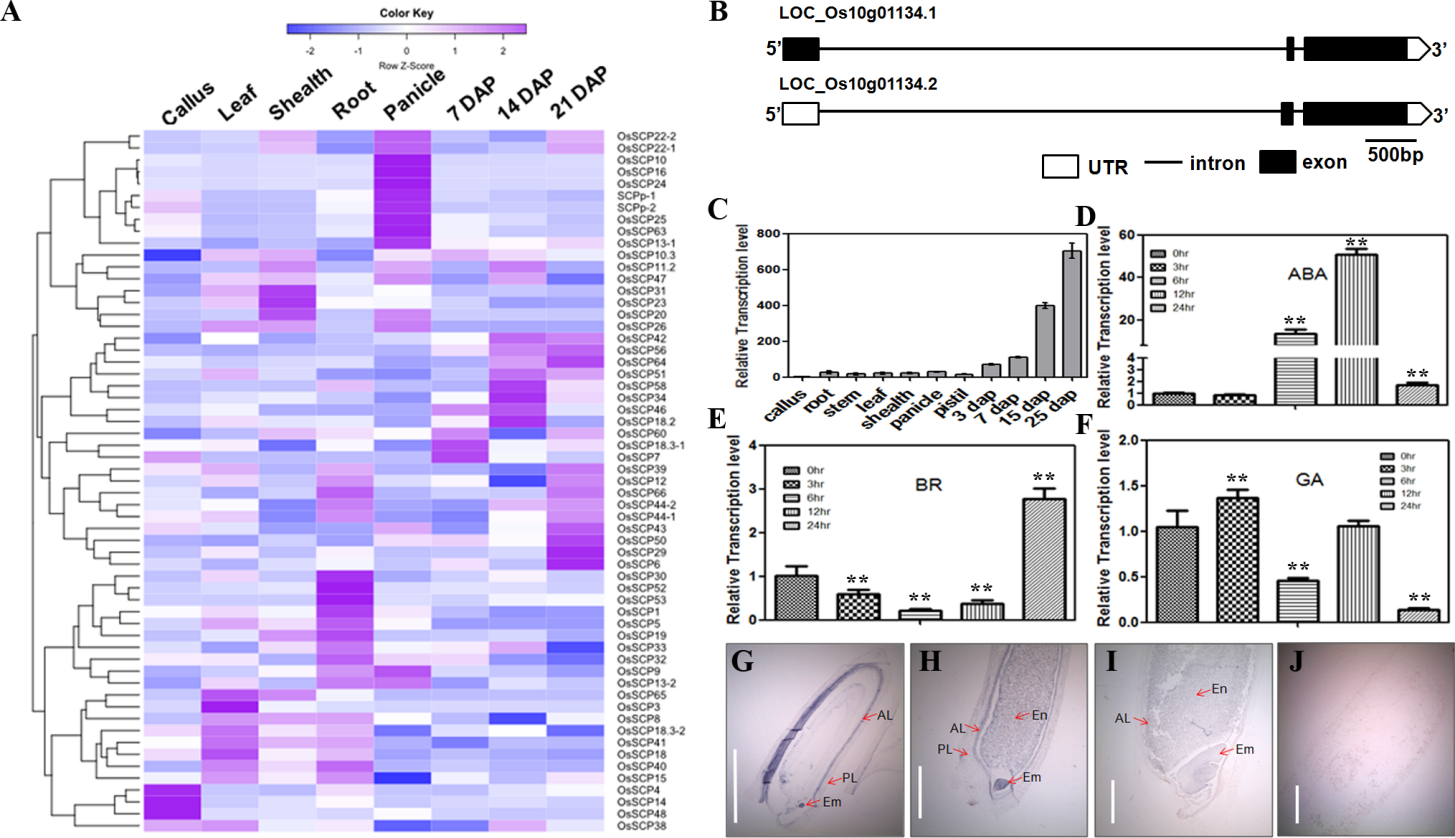
Expression analysis of *SCPs* in tissues and response to phytohormone treatments. (A) Hierarchical cluster display of the expression profile of rice *SCPs* in callus, leaf, shealth, root, panicle, 7, 14 and 21 DAP seeds. Color bar on the top represents the log2 expression values. Blue, white and Purple indicate the low, medium and high expression values respectively. (B) Schematical representation of the gene structure of *SCP46*. (C) qRT-PCR analysis of *SCP46* in various rice tissues. Different characters indicate a statistically significant difference at P<0.05. (D-F) qRT-PCR analysis of *SCP46* in response to ABA (D), BR (E) and GA (F) treatment at 5 different time points. ** indicates P<0.01 by t-test in comparison with 0h. (G-J) mRNA *in-situ* hybridization assay of *SCP46* in seeds at early (G), medium (H) and late stages (I); (J) shows the negative control by using sense probe for hybridization. Em: embryo; En: endosperm; PL: pericap layer; AL: aleuron layer; Bar = 1.5 mm.

A genome survey of the rice genome annotation project (RGAP) database (http://rice.plantbiology.msu.edu/) [32] resulted in the identification of 59 *SCP* genes in rice. By following the nomenclature of RGAP, all these SCPs were named from *OsSCP1* to *66* with some numbers skipped due to the updated genome annotation. Table 1 presents the comprehensive information of this rice gene family. The 59 *SCPs* are distributed in different loci of the 12 rice chromosomes in the form of a single gene or gene cluster. The gene length of *SCPs* varies from 600bp to 33 kb, while the protein lengths are in the range of 300 to 500 amino acids with a few extreme exceptions (Table 1). Additionally, we downloaded 40 Maize *SCPs* from the MaizeGDB (http://www.maizegdb.org/) [33]. To investigate the evolutionary relationship of rice and maize *SCP* genes, the amino acid sequences of all the 99 genes were used as an input for the construction of the SCP phylogenic map by using ClustalW (MEGA7.0). The results suggested that the SCPs are largely diversified (Fig 1). The maize 2G123815 was the closest ortholog to GS5, indicating that this gene could also be functionally a seed development regulator. On the other hand, we found that some clades barely contain any maize SCPs, implying that this subgroup is highly specified in rice. CREP (Collection of Rice Expression Profiles) (http://crep.ncpgr.cn/) collected the expression profiles of rice genes in 39 tissues from 2 elite varieties [34]. To gain an overall view of the *SCP* expression profiles, we downloaded the expression data in 8 typical tissues and stages from CREP including callus, leaf, sheath, root, panicle, 7 DAP endosperm, 14 DAP endosperm and 21 DAP endosperm, and displayed the profile as a heat map in Fig 2A. As a result, *SCP* genes showed divergent expression patterns. Some members such as *SCP11* and *26* were constitutively expressed in all the tissues. However, we did find several *SCPs* showed highly tissue specific expression patterns. For example, *OsSCP3* was dominantly expressed in leaf, while *OsSCP10*, *16* and *24* were specifically expressed in panicles. As the research focus in our lab is rice seed development, we also attempted to find out the seed-specific or -dominant *SCP* genes. It turned out that the expression level in endosperms was at least 4 folds higher than in other tissues for *SCP6*, *46* and *56*, which suggested a seed-dominant expression pattern and intrigued us to further explore their biological function in rice seed development. The divergent expression profiles of *SCPs* indicated versatile roles of this gene family in rice growth and development.

**Table 1:** Comprehensive information of *SCPs* in rice.

**Gene**	**Locus ID**	**Gene length (bp)**	**CDS length (bp)**	**Protein length (AA)**	**Domain length**	**Molecular weight (KDa)**	**PI**	**Exon**	**Intron**
OsSCP1	LOC_Os01g06490	2734	1416	471	412	52.53	5.2508	9	8
OsSCP2	LOC_Os01g11670	1686	1290	429	392	46.26	7.0798	2	1
OsSCP3	LOC_Os01g22980	4263	1442	480	427	54.32	6.6402	10	9
OsSCP4	LOC_Os01g43890	2066	1359	452	394	48.08	7.148	1	0
OsSCP5	LOC_Os01g61690	3631	1365	454	415	50.54	6.9461	9	8
OsSCP6	LOC_Os02g02320	4869	1503	500	399	55.45	6.0623	9	8
OsSCP7	LOC_Os02g26480	9003	1512	503	429	56.85	5.6285	14	13
OsSCP8	LOC_Os02g42310	7255	1458	485	427	53.92	6.3211	10	9
OsSCP9	LOC_Os02g46260	5212	1443	480	428	53.62	5.7362	14	13
OsSCP10	LOC_Os02g55130	7227	1422	473	427	52.34	9.3639	9	8
OsSCP11	LOC_Os03g09190	3522	1398	465	410	51.78	6.6214	6	5
OsSCP12	LOC_Os03g26920	2377	1512	503	425	55.96	4.7272	8	7
OsSCP13	LOC_Os03g26930	3430	1437	478	409	53.52	6.5811	8	7
OsSCP14	LOC_Os03g27480	3912	1413	470	414	50.98	7.5554	12	11
OsSCP15	LOC_Os03g27510	4805	1014	337	259	36.82	9.4207	11	10
OsSCP16	LOC_Os03g27530	6926	1173	390	291	42.84	6.7185	11	10
OsSCP17	LOC_Os03g27550	626	237	78	44	8.53	9.7602	3	2
OsSCP18	LOC_Os03g27590	5445	1413	470	424	50.79	4.7504	12	11
OsSCP19	LOC_Os03g52040	5692	1452	483	431	53.88	7.3067	11	10
OsSCP20	LOC_Os03g52070	7317	1317	438	384	48.56	6.9292	11	10
OsSCP21	LOC_Os03g52080	4148	1203	400	138	44.81	7.1324	13	12
OsSCP22	LOC_Os04g09720	8641	1404	467	422	51.69	6.0767	14	13
OsSCP23	LOC_Os04g25560	6011	1515	504	458	56.11	6.4622	14	13
OsSCP24	LOC_Os04g32540	3666	1413	470	422	51.45	6.5161	8	7
OsSCP25	LOC_Os04g44410	4606	1407	468	417	52.02	9.0621	7	6
GS5/OsSCP26	LOC_Os05g06660	4560	1452	483	428	53.73	6.509	10	9
OsSCP28	LOC_Os05g18604	33199	1452	483	419	53.77	7.1201	10	9
OsSCP29	LOC_Os05g50570	1574	1341	446	395	46.97	7.6887	1	0
OsSCP30	LOC_Os05g50580	1938	1356	451	400	47.76	6.4078	1	0
OsSCP31	LOC_Os05g50600	1473	1329	442	391	47.37	7.0264	1	0
OsSCP32	LOC_Os06g08720	7467	1455	484	419	53.41	5.6715	10	9
OsSCP33	LOC_Os06g13410	647	576	191	63	20.11	8.8431	2	1
OsSCP34	LOC_Os06g13420	2193	876	291	41	31.37	10.6293	4	3
OsSCP35	LOC_Os06g32740	4493	3273	1090	62	125.08	6.2913	6	5
OsSCP36	LOC_Os06g32770	2318	1593	531	438	59.14	7.7865	2	1
OsSCP37	LOC_Os06g36570	756	756	252	148	27.34	8.8198	1	0
OsSCP38	LOC_Os06g51370	2152	1503	501	431	55.48	6.5071	3	2
OsSCP39	LOC_Os07g29620	3827	1575	525	400	57.35	5.2912	9	8
OsSCP40	LOC_Os07g46350	4983	1575	525	423	56.15	5.4391	7	6
OsSCP41	LOC_Os08g44640	5339	1407	469	423	51.46	6.3128	12	11
OsSCP42	LOC_Os09g28830	3725	1335	445	337	49.06	8.6758	8	7
OsSCP43	LOC_Os09g28840	4851	1809	603	261	66.78	6.2419	11	10
OsSCP44	LOC_Os10g01110	3115	1383	461	410	51.34	7.1007	8	7
OsSCP46	LOC_Os10g01134	6317	1419	473	416	52.13	5.3897	3	2
OsSCP47	LOC_Os10g39560	3212	1383	461	389	51.27	5.4377	10	9
OsSCP48	LOC_Os11g10750	4046	1398	466	421	51.71	6.5081	10	9
OsSCP50	LOC_Os11g24180	15020	1377	459	403	51.07	5.2605	12	11
OsSCP51	LOC_Os11g24290	4028	690	230	164	25.27	4.4332	7	6
OsSCP52	LOC_Os11g24320	3720	486	162	57	17.55	4.5268	5	4
OsSCP53	LOC_Os11g24340	4668	1188	396	333	44.72	7.6011	9	8
OsSCP55	LOC_Os11g24374	24718	1182	394	347	43.87	6.5019	12	11
OsSCP56	LOC_Os11g24510	8005	1440	480	429	53.36	5.8082	13	12
OsSCP58	LOC_Os11g27170	19261	1425	475	400	52.96	5.4265	12	11
OsSCP60	LOC_Os11g27264	11827	1452	484	430	53.86	5.7842	14	13
OsSCP62	LOC_Os11g27329	31581	1077	359	258	39.52	5.6281	14	13
OsSCP63	LOC_Os11g31980	3853	1419	473	410	52.61	6.5171	10	9
OsSCP64	LOC_Os11g42390	6132	1389	463	369	51.35	7.9475	8	7
OsSCP65	LOC_Os12g15470	5108	1566	522	447	56.77	7.7368	14	13
OsSCP66	LOC_Os12g39170	6120	1362	454	402	50.48	5.9073	10	9

### *SCP46* is dominantly expressed in embryo, endosperm and aleurone layer of developing seeds

A spatial- or temporal-expression pattern usually indicates the critical roles of the genes in the corresponding biological process. According to the microarray data from CREP, *SCP46* is dominantly expressed in rice developing seeds. Due to the alternative splicing models, *SCP46* is predicted to have 2 isoforms, both of which consist of 3 exons and 2 introns (Fig 2B). To validate the expression profile of *SCP46*, we performed quantitative RT-PCR to examine the transcriptional level of *SCP46* in ten various rice tissues and stages. As shown in Fig 2B, the *SCP46* mRNA abundances were extremely low in callus, leaf, root, stem, old panicles and unpollinated pistils, but dominantly expressed in developing seeds. In particular, we found that the *SCP46* transcription level increased with the development of seeds, and reached a maximum level at 25 DAP, suggesting that *SCP46* may have an important function in rice grain filling and seed maturation. In addition, we checked the transcription level of *SCP46* in response to the application of 100μM ABA, 100μM GA and 10μM BR at 0, 3, 6, 12 and 24 hours respectively. Upon ABA treatment, the *SCP46* transcription level was unchanged at 3h, but sharply increased to 18 fold and 51 fold at 6h and 12h respectively, and then decreased back to a low level at 24h, when compared with the control (Fig 2D). Such an ABA-induced expression pattern strongly suggested that *SCP46* may be involved in ABA signaling in rice. On the contrary, we observed an opposite trend of *SCP46* in response to BR (Fig 2E). The BR treatment significantly repressed the gene transcription level at 6h and 12h while increased the level at 24h, which is in accordance to the antagonistic role of BR to ABA [35]. To our surprise, we did not observe any featured expression pattern by the GA induction (Fig 2F), though GA is also antagonistic to ABA, indicating that *SCP46* is functionally irrelevant to GA response.

To gain an in-depth view of the *SCP46* profile, we further conducted RNA *in-situ* hybridization experiment in developing seeds of Nipponbare at various stages. In the early developing seeds (2–3 DAP, 1–2 mm in length), *SCP46* was mainly detected in the embryo and the inner layer of the seed coat (Fig 2G). Signals were also observed on cellularized endosperm cells, but in a weaker manner than the embryo (Fig 2G). With the development of seeds (4–5 DAP, 3–4 mm in length), *SCP46* was strongly accumulated in the embryo, endosperm and particularly in the aleurone layers. However, only weak expression of *SCP46* was detected in other parts of the seeds such as the pericap layer (Fig 2H). This type of expression pattern extended to the later stage (10–15 DAP, 6-7mm in length), except that the *SCP46* signal gradually weakened as the stage progressed (Fig 2I). No signals were detected when a sense probe was used as a negative control (Fig 2J). Given that sucrose and other photosynthesis assimilates are transported through the aleurone layers to the embryo sac cavity [36], a high expression in aleurone layer suggested that *SCP46* may be involved in rice grain filling.

### Knock-down of *SCP46* affected rice grain size and seed germination

**Fig 3:**
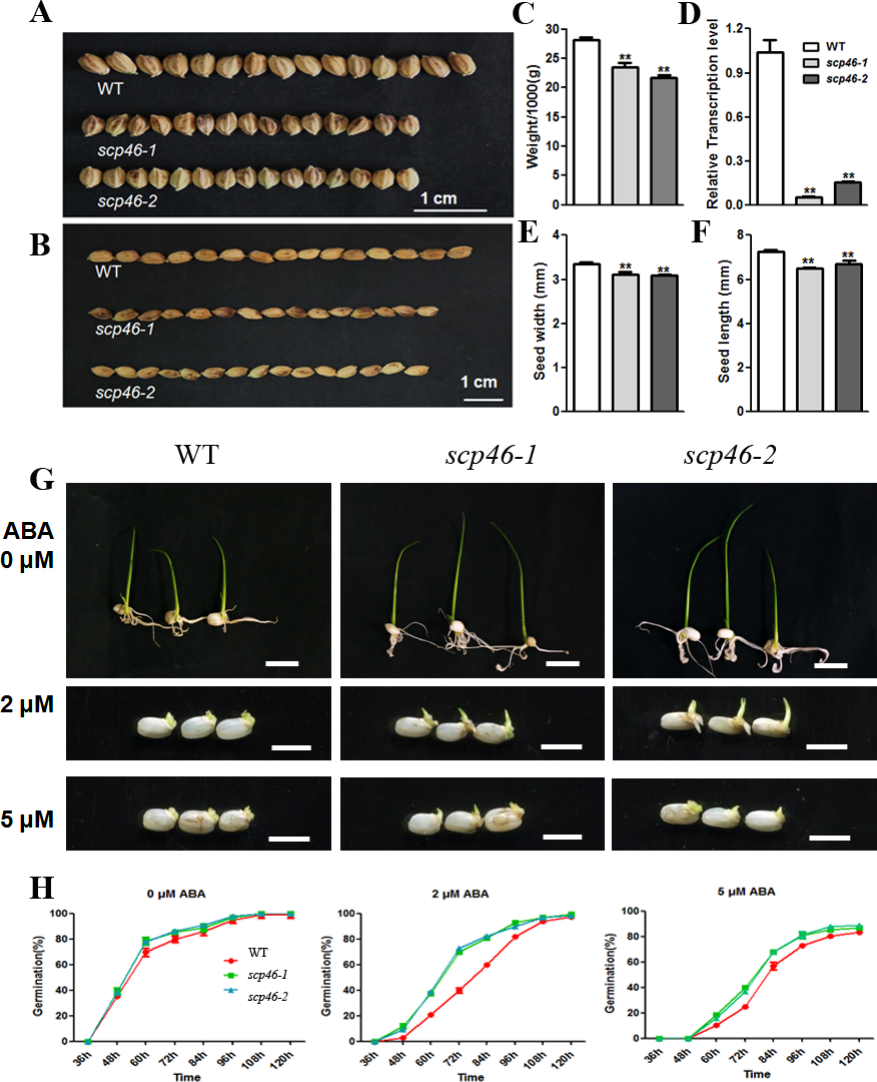
Phenotypical characterization of *scp46* grain size and seed germination. (A-B) Comparison of the width (A) and length (B) of *scp46* and WT grains; (C) qRT-PCR analysis of *SCP46* expression level in the seeds of RNAi and WT lines. 1000-grain-weight (D), grain length (E) and width (F) of RNAi and WT lines. (G) Seed germination of *scp46* and WT seeds under 0, 2 and 5 μM ABA. Pictures were taken at 120 hours after the germination assay. (H) Germination time courses of *scp46* and WT seeds. Asterisks indicate the significance of differences between Wild-type and *scp46* lines as determined by Student’s t test analysis: ** P<0.01.

To dissect the biological roles of *SCP46* in rice seed development, we generated 10 independent *SCP46* RNAi suppressing lines in the background of Kitaake (*Oryza sativa*, *ssp*.*japonica*), of which 6 lines showed substantial reduction of *SCP46* and similar phenotype in T_0_ generation (S1 Fig). For a better description of the plant phenotypes, the data of two representative T_0_ lines *scp46-1* and *scp46-2* are presented here. Our qRT-PCR results showed that the *SCP46* expression levels were significantly reduced to 5% and 15% of the wild-type plants respectively, suggesting a substantial reduction of the gene (P<0.01) (Fig 3C). Through a whole-life-cycle phenotype characterization, no obvious phenotypes were observed in the RNAi lines, except that both *scp46-1* and *scp46-2* showed significant reduction of grain size when compared with the wild-type. Under our growth conditions, the grain length of Kitaake was about 7.28 mm, whereas the grain length of *scp46-1* and *scp46-2* decreased to 6.51mm and 6.7mm respectively (Fig 3B and 3E). Meanwhile, the grain width of *scp46-1* and *scp46-2* also had a 7% and 8.4% reduction when compared with wild-type (Fig 3A and 3F). As the consequence of the smaller grain size, the 1000-grain-weights of RNAi lines were only 77.0–83.6% of that of the wild-type (Fig 3D). Furthermore, the high expression in embryo as well as the ABA-induced expression pattern intrigued us to test the function of *SCP46* in seed germination. In the MS medium with or without ABA added, both the seeds of *scp46-1* and *scp46-2* exhibited faster germination. When there is no ABA added in the MS medium, 79.7% of the wild-type seeds germinated in 72 hours, whereas 85.3% and 86.3% of the seeds were able to germinate for *scp46-1* and *scp46-2* respectively at the same time point (Fig 3G and 3H). The difference was further magnified when ABA was added into the medium. Under the 2 μM ABA treatment, there were 29.6% and 32.7% more of the *scp46* seeds were germinated than the wild-type at 72 hours. The 5 μM ABA treatment experiment exhibited a similar result as 2 μM ABA, though it took a longer time for all the seeds to germinate (Fig 3G and 3H). The enhanced germinability of *SCP46* RNAi seeds suggested that this gene may be involved in ABA signaling, and knock-down of *SCP46* repressed the rice sensitivity to ABA. In the following T1 generation, we consistently observed the similar phenotypes of *SCP46* RNAi seeds for 10 tested lines, thus we concluded that these phenotypes were attributed to the knock-down of *SCP46* (S2 Fig).

### *SCP46* regulates grain filling and seed germination related genes

To find out the genes regulated by *SCP46*, RNA-seq experiment was performed for the Wild-type and *scp46*-1 developing seeds at 7 DAP by using Illumina HiSeqTM 2000 platform. For Wild-type and *scp46-1*, 14,244,606 and 12,086,473 high quality (Q30>92%), clean reads were generated respectively, out of which 93.9% and 93.8% could be mapped into the rice genome. FPKM (Fragments Per Kilobase of transcript per Million fragments mapped) was employed to evaluate the gene transcriptional abundance. As a result, a total of 1835 genes were found to be differentially expressed between the RNAi plant and Wild-type, including 808 genes which showed over 2 fold up-regulation and 1027 genes were down-regulated in *scp46-1* (|log 2Ratio|≥1; FDR<0.001) (S1 Table). Among these DEGs (Differentially Expressed Genes), several have been known to be functionally relevant to rice seed development. For example, *SPK* (*Suc synthase Protein Kinase*) (LOC_Os10g39420), *OsGLN1* (*Glutamine synthesiase I*) (LOC_Os03g12290), *OsPPDKA* (*Pyruvate orthophosphate dikinase A*) (LOC_Os03g31750) and *OsEIN2 (Ethylene Insensitive 2)* (LOC_Os07g06130) are important regulator of rice grain filling, while *VP1* (*Viviparous 1*) (LOC_Os01g68370), *OsRACK1* (*Receptor for activated C kinase 1*) (LOC_Os01g53294) and *OsMT2b* (*Metallothionein2b*) (LOC_Os05g02070) are involved in the control of seed germination. A KEGG (Kyoto Encyclopedia of Genes and Genomes) pathway analysis of the DEGs revealed that high ratios of our DEGs were involved in the plant hormone and signal transduction, ribosome and various metabolism pathways (S3 Fig). In particular, we found that 12 DEGs are related to starch and sucrose metabolism, and 20 DEGs are related to photosynthesis antenna protein complex or photosynthesis process (S3 Fig). Given that photosynthesis and starch metabolism are two of the key processes for grain filling, the KEGG pathway result strongly supported our conclusion that *SCP46* regulates grain filling in rice.

**Fig 4:**
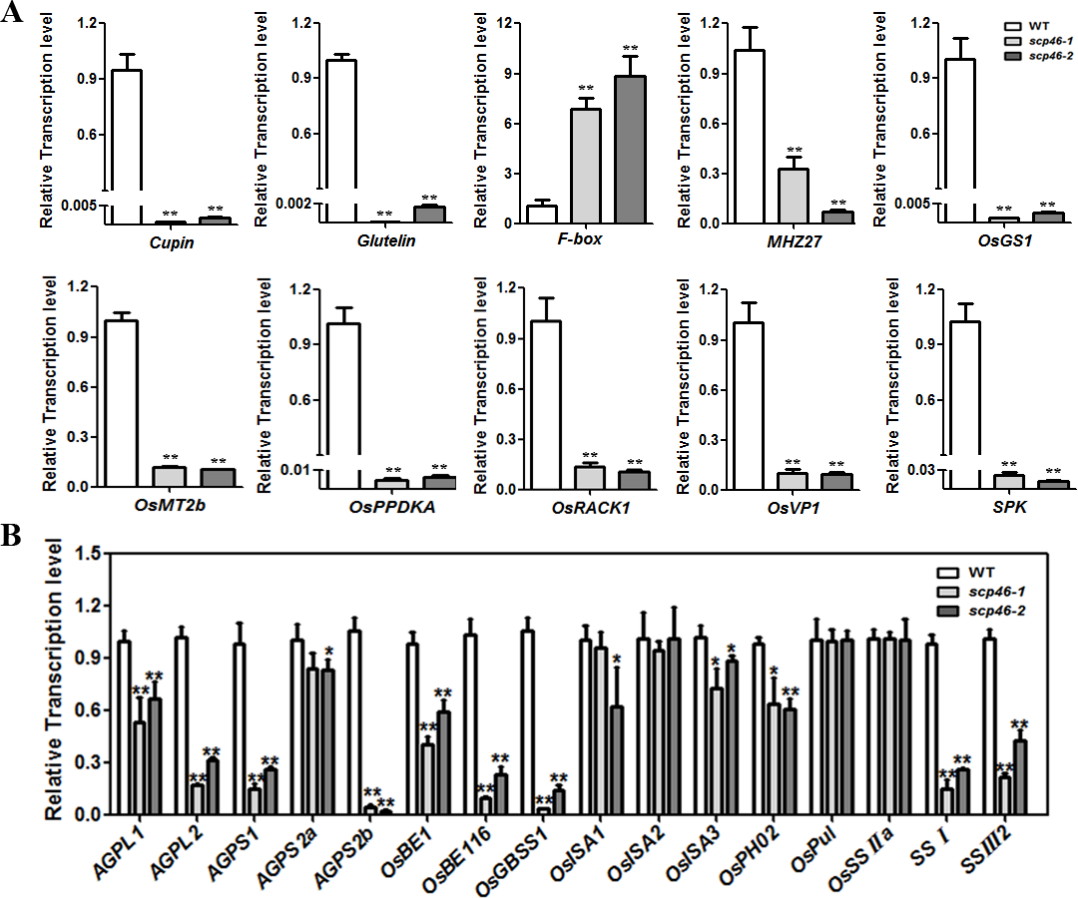
Expression analysis of *SCP46* regulated genes by qRT-PCR. (A) qRT-PCR validation of the DEGs revealed by RNA-seq experiments; (B) the expression change of rice grain filling genes in *scp46* and WT. 7 DAP seed cDNA was used as templates for this analysis. All values are based on three technical repeats and presented as means±SE.* indicates P<0.05, ** indicates P<0.01 by t-test.

To validate the RNA-seq data, 10 DEGs were selected for qRT-PCR verification. As shown in Fig 4A, the qRT-PCR result exhibited a similar tendency in the regulation pattern with the RNA-seq experiments, though the extent of expression change may slightly vary from gene to gene, suggesting that the RNA-seq data is highly reliable in this study. Given that RNA-seq results sometimes may ignored the DEGs with minor but statistically significant differences, we also examined the mRNA abundance of 16 other reported grain filling related genes which were not considered differentially expressed in DEG analysis except *OsPHO2*. These genes included rice starch synthesis rate limiting enzyme genes *AGPs (ADP-glucose pyrophosphorylase)*, *starch synthesases*. As a result, 11 of the tested genes were found to be down-regulated in both *scp46-1* and *scp46-2*, which well explained the reduced grain size in *scp46* plants (Fig 4B).

### SCP46 interacts with Dehydration-Induced 19–1 in yeast

**Fig 5:**
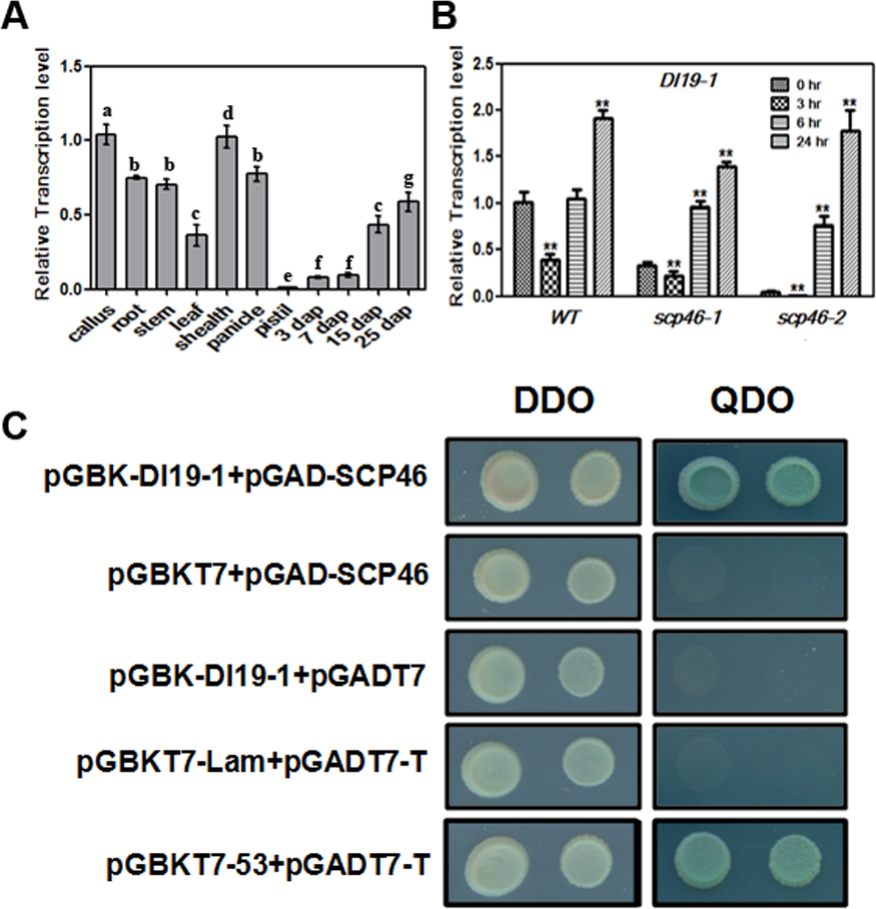
Expressoin profile of *DI19-1* and yeast-two-hybrid assay of SCP46-DI19-1 interaction. (A) Expression profile of *DI19-1* in different rice tissues; Different characters indicate a statistically significant difference at P<0.05. (B) SCP46 expression in response to ABA treatment; * indicates P<0.05, ** indicates P<0.01 by t-test in comparison with 0h. (C) yeast-two-hybrid assay of SCP46-DI19-1 interaction. pGBKT7-Lam+pGADT7-T and pGBKT7-53+pGADT7-T were used as negative and positive controls as instructed by the Kit. DDO: double drop out medium (SD/-Trp-Leu); QDO: quarter drop out medium (SD/-Trp-Leu-Ade-His/+X-α-Gal).

To search for the SCP46 interactive proteins, the full coding sequence of *SCP46* was fused with GAL4 DNA-BD domain, and used as a bait to screen a cDNA expression library constructed from Nipponbare young seedling mRNA. A total of 8 million colonies were screened and resulted in the identification of 9 unique SCP46 interactive proteins (Table 2). Although the 9 proteins are functionally unknown, the DI19-1 (Dehydration-Induced 19–1) protein attracted our interest. DI19 is a group of transcription factors containing two unusual, but evolutionally conserved Cys2-His2 (C2H2) putative zinc finger domains [37]. DI19 proteins are responsive to various abiotic stresses like dehydration, heat and salinity. Though Arabidopsis DI19s are not induced by ABA, the members in cotton, maize and rice have been reported to be involved in ABA signaling [38–40]. DI19-1 was constitutively expressed in all the tested tissues. However, we found that the expression of *DI19-1* gradually increased with the development of seeds and reached a plateau in 25 DAP (Fig 5A), which is similar to the tendency of *SCP46*. On the other hand, we attempted to examine the *DI19-1* expression in Wild-type and *scp46* lines in response to ABA treatment. In the Wild-type, *DI19-1* was induced by ABA treatment at 24 hours, indicating a role of it in ABA signaling as well. Meanwhile, *DI19-1* displayed a lower expresion level at each time point in *scp46s*, but very similar expression tendencies as in the Wild-type, suggesting that Knock-down of *SCP46* down-regulated the expression of *DI19-1*. To confirm the SCP46-DI19-1 interaction in yeast, we did a reciprocal hybridization by fusing SCP46 with AD and DI19-1 with BD domain respectively. As we expected, yeast co-transformed with pGBK-DI19-1 and pGAD-SCP46 could grow on the quart drop-out medium as the positive control did. Meanwhile, no colonies were visible in the selection medium for all the negative controls, suggesting the interaction between SCP46 and DI19-1 is reliable and specific in yeast.

**Table 2:** Interactive proteins of SCP46 identified by yeast two hybrid assay.

**Locus ID**	**Protein annotation**
LOC_Os02g06890	ubiquitin thioesterase OTU1
LOC_Os02g10190	uncharacterized protein
LOC_Os05g05950	translocase of chloroplast 159
LOC_Os05g09740	acid phosphatase 1
Os05g0334400	chaperone protein DnaJ
LOC_Os05g48800	Dehydration-induced 19–1
LOC_Os06g47890	adagio-like protein 1
LOC_Os07g01990	uncharacterized protein
LOC_Os09g03610	flowering time control protein FCA

All the interactive protein annotation was extracted from RGAP (http://rice.plantbiology.msu.edu/)

## Discussion

Proteolysis is not only a key mechanism to maintain the balance of protein synthesis and degradation, but also a major type of post-translational modification for functional protein maturation by cleaving amino acids off the protein precursors, thus altering the protein structures or biochemical activities [11]. Serine proteases are one of the largest groups of proteolytic enzymes found in plants and are associated with several essential physiological pathways. In rice, one the most important food crop in the world, at least 59 *SCPs* were found with detailed gene structures information in this study. Unfortunately, the function of only a few *SCPs* has been characterized till now. A well-documented example is that *GS5/OsSCP26* was found to be a major QTL regulating grain width, filling and weight [31]. Sequence association analysis of the *GS5* promoter region revealed three haplotypes controlling the grain width in 51 rice accessions from a wide geographic range, which suggested that natural variation in *GS5* contributes to grain size diversity in rice. A followed up study further demonstrated that 2 SNPs (Single Nucleotide Polymorphisms) in upstream of the gene elevated the ABA-induced expression of effective *GS5* in developing young panicles, promoted the mitotic division in lemma/palea *via* a BR signaling pathway, and eventually resulted in wider grains [41]. Despite the understanding of *GS5*, the function of the rest of the *OsSCPs* other than *GS5* remains untouched. Generally, two strategies have been routinely used to predict the roles of unknown genes. One is to establish a phylogenic relationship of the candidate genes with other potential gene family members by amino acid sequence alignment, because the knowledge from homologs or orthologs from other related species are usually applicable in the candidate genes. Interestingly, we noticed that the subgroup where SCP46 located in consists 17 rice proteins but only one maize protein (2G011784). The expansion of rice members suggests more complicated functions of these SCPs in rice than maize. Meanwhile, the close phylogenetic relationship between SCP46 and 2G011784 also implies similar function of 2G011784 in maize seed development. Another way is to gain clues from the temporal- or spatial-expression profiles, as a featured expression pattern is usually indicative of the gene function in certain biological processes. Due to the limited knowledge of *SCPs* in plants, we relied more on the expression profile strategy to start our research on *OsSCPs* in this study. Interestingly, several *OsSCPs* displayed tissue-specific or dominant patterns, including *OsSCP3* which is dominantly expressed in leaf, *OsSCP10*, *16* and *24* which are specifically expressed in panicle, and *SCP6*, *46* and *56* which are dominantly accumulated in developing seeds, suggesting crucial roles of these SCP genes in the development of corresponding tissues. Besides the tissue profile, examining the expression profile of *OsSCPs* under different hormone treatments or in various biological processes would be very helpful for their functional characterization.

In this study, a reverse genetic strategy starting from the tissue-specific expression pattern was employed to study the functions of *OsSCPs*. The microarray data together with our qRT-PCR results both clearly showed that *SCP46* is dominantly expressed in developing seeds, especially in the late stages. Moreover, the RNA *in-situ* hybridization emphasized the accumulation of *SCP46* in embryo, endosperm and aleurone layers. It is therefore rational for us to put our research focus of *SCP46* function in rice grain filling. Not surprisingly, the grain size was significantly reduced when *SCP46* was suppressed. The consistent phenotype observed on multiple independent RNAi lines for two consecutive generations strongly indicated that *SCP46* controls rice grain size possibly *via* regulation of the grain fillings. Our conclusions were also supported by the RNA-seq result, in which several key coordinators of grain filling, such as *SPK* (LOC_Os10g39420), *OsEIN2* (LOC_Os07g06130), *OsGLN1* (LOC_Os03g12290) and *OsPPDKA* (LOC_Os03g31750) were down-regulated in *scp46* lines [42–45]. *SPK* (LOC_Os10g39420) encoding a calmodulin-like domain protein kinase was 6.3 fold down-regulated in *scp46-1*. *SPK* is specifically expressed in the endosperm of immature seeds. Antisense *SPK* transformants produced defected seeds with no starch accumulated. More evidences showed that *SPK* controls the activity of sucrose synthase by catalyzing phosphorylation at the conserved RxxS domain, hence to regulate sucrose synthesis, which is the initial step for the biosynthesis of storage starch [44]. *OsEIN2* is another down-regulated gene in *SCP46* RNAi plants. *OsEIN2* participates in ethylene signaling. Grain length was promoted in *OsEIN2*-transgenic plants and 1000-grain weight was reduced in *osein2* mutants [45]. *OsGLN1* encodes a cytosolic glutamine synthetase. It was reported that *OsGLN1* regulates rice nitrogen and carbon assimilation, which finally affects the plant growth rate and grain filling [42]. Literature also described that regulatory phosphorylation of *OsPPKDA* might be involved in the rice seed development [43]. In addition to the regulators, rice starch synthesis genes and storage protein genes were affected by *SCP46* as well. Our RNA-seq DEG analysis together with the qRT-PCR results identified at least 9 glutelin genes, 5 cupin genes and 16 late embryogenesis genes for storage proteins synthesis. Meanwhile, all the 5 *AGP* complex members, which are the rate limiting enzymes of starch synthesis, were repressed in *SCP46* RNAi plants too.

Because of the high accumulation in the embryo and inducible pattern to ABA treatment of *SCP46*, we attempted to observe the performance of *SCP46* RNAi seeds during germination. In pure MS medium, *scp46* seeds showed weaker dormancy than the Wild-type. Accordingly, we found that *VP1* (LOC_Os01g68370) and *OsRACK1A* (LOC_Os01g53294) were repressed by the knock-down of *SCP46*. VP1 has been known to regulate seed maturation and dormancy by interacting with TRAB1 and mediating ABA-induced transcription [46]. Meanwhile, *OsRACK1A* positively regulates seed germination and negatively regulates the responses of seed germination to exogenous ABA, which is similar to the phenotype observed in *OsSCP46* RNAi seeds [47]. Moreover, the germinability differences between *scp46* and Wild-type seeds were further magnified when ABA was applied to the medium, strongly indicating that *SCP46* is involved in the ABA signaling, and the enhanced germination was due to the reduced ABA sensitivity. To detect proteins interacting with SCP46, we did yeast-two-hybrid screening and found an ABA-inducible protein DI19-1 could bind with SCP46. However, though both proteins shared similar ABA-induced pattern, it is unlikely that DI19-1 acts as a substrate of SCP46 proteolysis because *DI19-1* was found to be down-regulated in the two *scp46* lines as revealed by qRT-PCR (Fig 5B). Presumably, DI19-1 works with SCP46 and other unknown factors as a protein complex in proteolysis in seed development.

ABA is an isoprenoid hormone playing critical roles in various plant biological processes, including grain filling and germination [48]. Early in 1993, scientists found that ABA could control the rate of assimilate accumulation from source organ to sink organ by promoting unloading of assimilates from sieve tube to sink apoplast and uptake of unloaded assimilates by storage cells [49]. However, the effects of ABA on grain filling are dosage-dependent, as also numerous literatures described that high concentration and hypersensitivity of the ABA could inhibit grain fillings and lead to smaller seeds [50,51]. On the other hand, ABA has been well-known for its critical roles in seed dormancy and germination [52,53]. Disruption of maize ABA signaling elements *VP1* gives rise to pre-harvest sprouting (PHS) [54]. PHS in modern hexaploid wheat varieties and the sprouting susceptible rice varieties was ascribed to the missplicing of wheat *Vp1* genes and rice *Vp1* counterpart [55,56]. It was also reported that the mutant of *ABI3*, the close ortholog of *VP1* in Arabidopsis, showed insensitivity to ABA, reduced grain filling and activated germination, which mimicked the phenotype of *vp1* and *scp46* observed in this study [57,58]. More interestingly, it happened that rice *VP1* was significantly down-regulated in the *scp46* as indicated by both DEG and qRT-PCR results. Given the down-regulation of *OsVP1* and similar phenotype observed in Arabidopsis, we proposed that *OsVP1* may be involved in the *SCP46*–regulated rice grain filling and seed germination.

## Materials and Methods

### Sequence alignments and phylogenetic analysis

The information regarding the *SCP* genomic sequence, CDS sequence and protein sequence was downloaded from the Rice Genome Annotation Project (http://rice.plantbiology.msu.edu/). The sequence of maize SCPL gene family was downloaded from MaizeGDB (http://www.maizegdb.org/). The numbers of the SCPL gene family were analyzed by ClustalW (MEGA 7.0). The phylogenetic tree was constructed in MEGA7.0 using the Maximum Likelihood. The evolutionary distances were computed using the Jones-Taylor-Thornton (JTT) model, and the substitutions type was amino acid. There were about 1,000 replicates performed in each analysis in the bootstrap test to obtain confidence support. The ML tree was searched using the Nearest-Neighbor-Interchange (NNI). The branch length scale bar indicates the evolutionary distance.

### Plant materials and growth condition

Nipponbare, Kitaake (*Oryza sativa*, *ssp japonica*) and all the transgenic plants used in this study were grown in the experimental field of China National Rice Research Institute in the summer of 2014 and 2015. The callus, root, stem, leaf, sheath, pistil and seeds in different developmental stages of Nipponbare, and 7 DAP seeds in *SCP46* RNAi and Kitaake were put into liquid nitrogen immediate after harvest and stored at -80°C for total RNA isolation. For the phytohormone treatment, 14-day-old Nipponbare young seedlings with intact roots were kept in a beaker with water containing 100μM ABA (Sigma, St Louise, U.S.A.), 100μM GA (Sigma, St Louise, U.S.A.) and 10μM BR (Sigma, St Louise, U.S.A.) respectively, the whole seedling samples were collected at the corresponding time points and immediately used for RNA isolation. ABA treatment on 14-day-old Kitaake and *scp46* young seedlings was conducted by the same method as above.

### RNA *in-situ* hybridization

Nipponbare seeds at different developmental stages were fixed in diethyl pyrocarbonate (DEPC)-treated 70% FAA fix solution (70% ethanol, 5% glacial acetic acid and 3.7% formaldehyde) overnight at 4°C, then dehydrated through a series of ethanol solutions, infiltrated with xylene and embedded in paraffin. The embedded tissues were cut into 9-μm-thick sections. The *SCP46* probe was PCR amplified by primer set In-SCP46F+In-SCP46R. After being cloned into pGEM-T Easy vector (Promega, Madison, U.S.A.), the segmental sequence was transcribed *in vitro* to synthesize sense and antisense probes from either T_7_ or SP_6_ promoter using the Digoxigenin RNA labeling kit (Roche, Basilea, Swiss Confederation). The sense strand was used as a negative reference. The hybridization and immunological detection were conducted as described by *Zhang et al*.*2010* [59].

### Vector construction

To construct the *SCP46* RNAi construct, a 300 bp-long, unique segment of *SCP46* coding sequence was amplified by the primer set SCP46RNAif and SCP46RNAir. The PCR fragment was firstly cloned into pENTR/D by TOPO cloning kit (Invitrogen, Carlsbad, U.S.A.). The intermediate vector was then linearized by EcoRV cut and finally cloned into pANDA vector by attL×attR recombination reaction [60].

For the *SCP46* bait construct, the entire *SCP46* CDS fragment was amplified by PCR using *BamHI* and *SalI* linker primers and cloned into vector pGBKT7 by ligation. The *DI19-1* CDS fragment was amplified by PCR using *BamHI* and *EcoRI* linker primers, then digested and linked with the pGADT7 vector. All the primers used are listed in S2 Table.

### Phenotypic analysis of rice transgenic plant seeds

The length, width and thousand-grain-weight of WT and *scp46* lines were examined by a seed phenotyping system (Wangsheng, Hangzhou, China), and the data was analyzed by student *t-test* in Microsoft Office Excel 2010. For the seed germination assay, Kitaake and *scp46* seeds were firstly surface sterilized by washing in 70% ethanol for 1 minutes and 50% bleach for 30 minutes. After being flushed with autoclaved water for several times, the seeds were placed on Murashige and Skoog medium containing 0, 2 and 5 μM ABA respectively, and kept in a growth chamber (28°C, 14 hours daytime and 10 hours night time). Seeds which formed green shoots were considered as germinated. Seed germination was scored every 12 hours for 5 days. For each medium with different ABA concentration, at least 300 seeds (100 seeds with three replicates) of each line were tested. PCR with a pair of hygromycin primers was conducted for all the seedlings to exclude the T-DNA negative lines for the germination assay.

### RNA-seq

7 DAP rice seed total RNA was analyzed by Nanodrop 2000 spectrophotometer and Agilent 2100 Bioanalyzer to assure the proper purity, concentration and integrity. The qualified RNA was preceded for sequencing library construction as described by Hou et al (2015) [61]. The high-throughput sequencing was performed using the Illumina HiSeq™ 2000 platform when the quality of library was up to standard. The generated clean reads were aligned with reference sequences of rice in RGAP (http://rice.plantbiology.msu.edu/). Gene expression changes between the samples were analyzed by SOAP aligner/SOAP2 software. The KEGG pathway analysis of the DEGs was ultimately done by Biomarker technologies Co., Ltd., Beijing, China.

### RNA isolation and qRT-PCR

The RNA of all the tissues except developing seeds was extracted by Trizol (Invitrogen, Carlsbad, U.S.A.) according to the manufacturer's instructions. For developing seeds RNA extraction, a modified SDS-trizol method was applied as described by an online protocol (http://www.ag.arizona.edu/research/larkinslab/protocols/RNA%20extraction%20from%20endosperm%20-SDS-Trizol%20combo.pdf). Briefly, 0.5 grams of grinded seed powders were treated with 300 μL SDS RNA extraction buffer (50mM Tris.Cl pH 8.0, 5mM EDTA pH 8.0, 150mM LiCl and 1% SDS), 300 μL Phenol (pH 8.0): Chloroform = 1:1, the RNA in supernatant was then extracted by Trizol as for other tissues. The total RNA was quantified by Nanodrop spectrometer and 2 μg of each was used for reverse transcription by using M-MLV reverse transcriptase (Takara) with oligo(dT)_20_ (Invitrogen, Carlsbad, U.S.A.) as primer. A total reaction volume of ten microliter (5 μL THUNDERBIRD SYBR® qPCR Mix [Toyobo], 1 μL cDNA, 0.2μL primers, and 3.8μL water) was performed on the CFX96 touch realtime PCR detection system (Bio-rad, Hercules, U.S.A.) with three technical replicates. Expression was assessed by evaluating threshold cycle (CT) values. The relative expression level of tested genes was normalized to ubiquitin gene and calculated by the 2-^ΔΔ^CT method [62]. The sequence of all the primers used in this study can be found in S2 Table.

### Yeast-two-hybrid assay

The Matchmaker™ Gold Yeast Two-Hybrid system (Clontech, Dalian, China) was used to perform the yeast two-hybrid experiment. The rice young seedling cDNA library in Y2HGold strain was purchased from Clontech. Both the bait vector was transformed into yeast Y187 strain and mated with cDNA library according to the manufacturer’s instructions. The SCP46-DI19 yeast two hybrid assay was conducted by co-transformation of both plasmids into Y2HGold. The co-transformed yeast cell was cultured in SD/-Trp-Leu solid medium, and the interaction was confirmed by the colony growth in SD/-Trp-Leu-Ade-His solid medium with X-α-Gal.

## Supporting Information

S1 Fig The expression level of *SCP46* and seed weight in T_0_ RNAi lines. (TIF)

S2 Fig The expression level of *SCP46* and seed weight in T_1_ RNAi lines. (TIF)

S3 Fig DEG distribution in Enriched KEGG pathways. (TIF)

S1 Table DEGs between *scp46* and WT. (XLSX)

S2 Table Primers used in this study. (XLS)

## References

[1] Hayashi R, Moore S, Stein WH (1974) Serine at the active center of yeast carboxypeptidase. Journal of Biological Chemistry 248: 8366–8369. 4587122

[2] Feng Y, Yu C (2009) Genome-wide comparative study of rice and Arabidopsis serine carboxypeptidase-like protein families. Journal of Zhejiang University 35: 1–15.

[3] Breddam K (1986) SERINE CARBOXYPEPTIDASES. A REVIEW. Carlsberg Res Commun 51: 83–128.

[4] Sapio MR, Fricker LD (2014) Carboxypeptidases in disease: insights from peptidomic studies. Proteomics Clin Appl 8: 327–337. 10.1002/prca.201300090 24470285 PMC4062080

[5] Oda K (2012) New families of carboxyl peptidases: serine-carboxyl peptidases and glutamic peptidases. J Biochem 151: 13–25. 10.1093/jb/mvr129 22016395

[6] Galjart NJ, Gillemans N, Harris A, van der Horst GT, Verheijen FW, Galjaard H, et al (1988) Expression of cDNA encoding the human "protective protein" associated with lysosomal beta-galactosidase and neuraminidase: homology to yeast proteases. Cell 54: 755–764. 3136930 10.1016/s0092-8674(88)90999-3

[7] Fraser CM, Rider LW, Chapple C (2005) An expression and bioinformatics analysis of the Arabidopsis serine carboxypeptidase-like gene family. Plant Physiol 138: 1136–1148. 15908604 10.1104/pp.104.057950 PMC1150427

[8] Feng Y, Xue Q (2006) The serine carboxypeptidase like gene family of rice (Oryza sativa L. ssp. japonica). Funct Integr Genomics 6: 14–24. 15809843 10.1007/s10142-005-0131-8

[9] Jones CG, Lycett GW, Tucker GA (1996) Protease inhibitor studies and cloning of a serine carboxypeptidase cDNA from germinating seeds of pea (Pisum sativum L.). Eur J Biochem 235: 574–578. 8654403 10.1111/j.1432-1033.1996.00574.x

[10] Wajant H, Mundry KW, Pfizenmaier K (1994) Molecular cloning of hydroxynitrile lyase from Sorghum bicolor (L.). Homologies to serine carboxypeptidases. Plant Mol Biol 26: 735–746. 7948927 10.1007/BF00013758

[11] Tripathi LP, Sowdhamini R (2006) Cross genome comparisons of serine proteases in Arabidopsis and rice. BMC Genomics 7: 200 16895613 10.1186/1471-2164-7-200 PMC1560137

[12] Mugford ST, Osbourn A (2010) Evolution of serine carboxypeptidase-like acyltransferases in the monocots. Plant Signal Behav 5: 193–195. 20173416 10.4161/psb.5.2.11093 PMC2884133

[13] Dal Degan F, Rocher A, Cameron-Mills V, von Wettstein D (1994) The expression of serine carboxypeptidases during maturation and germination of the barley grain. Proc Natl Acad Sci U S A 91: 8209–8213. 7520177 10.1073/pnas.91.17.8209 PMC44575

[14] Wen J, Li J, Walker JC (2012) Overexpression of a serine carboxypeptidase increases carpel number and seed production in Arabidopsis thaliana. Food and Energy Security 1: 61–69.

[15] Li J, Lease KA, Tax FE, Walker JC (2001) BRS1, a serine carboxypeptidase, regulates BRI1 signaling in Arabidopsis thaliana. Proc Natl Acad Sci U S A 98: 5916–5921. 11320207 10.1073/pnas.091065998 PMC33313

[16] Domínguez F, González M, Cejudo FJ (2002) A germination-related gene encoding a serine carboxypeptidase is expressed during the differentiation of the vascular tissue in wheat grains and seedlings. Planta 215: 727–734. 12244437 10.1007/s00425-002-0809-2

[17] Cercos M, Urbez C, Carbonell J (2003) A serine carboxypeptidase gene (PsCP), expressed in early steps of reproductive and vegetative development in Pisum sativum, is induced by gibberellins. Plant Mol Biol 51: 165–174. 12602875 10.1023/a:1021142403856

[18] Bienert MD, Delannoy M, Navarre C, Boutry M (2012) NtSCP1 from tobacco is an extracellular serine carboxypeptidase III that has an impact on cell elongation. Plant Physiol 158: 1220–1229. 10.1104/pp.111.192088 22214816 PMC3291266

[19] Mugford ST, Milkowski C (2012) Serine carboxypeptidase-like acyltransferases from plants. Methods Enzymol 516: 279–297. 10.1016/B978-0-12-394291-3.00006-X 23034234

[20] Milkowski C, Strack D (2004) Serine carboxypeptidase-like acyltransferases. Phytochemistry 65: 517–524. 15003414 10.1016/j.phytochem.2003.12.018

[21] Schaller A (2004) A cut above the rest: the regulatory function of plant proteases. Planta 220: 183–197. 15517349 10.1007/s00425-004-1407-2

[22] Lehfeldt C, Shirley AM, Meyer K, Ruegger MO, Cusumano JC, Viitanen PV, et al (2000) Cloning of the SNG1 gene of Arabidopsis reveals a role for a serine carboxypeptidase-like protein as an acyltransferase in secondary metabolism. Plant Cell 12: 1295–1306. 10948250 10.1105/tpc.12.8.1295 PMC149103

[23] Mugford ST, Qi X, Bakht S, Hill L, Wegel E, Papadopoulou K, et al (2009) A serine carboxypeptidase-like acyltransferase is required for synthesis of antimicrobial compounds and disease resistance in oats. Plant Cell 21: 2473–2484. 10.1105/tpc.109.065870 19684243 PMC2751944

[24] Milkowski C, Baumert A, Schmidt D, Nehlin L, Strack D (2004) Molecular regulation of sinapate ester metabolism in Brassica napus: expression of genes, properties of the encoded proteins and correlation of enzyme activities with metabolite accumulation. Plant J 38: 80–92. 15053762 10.1111/j.1365-313X.2004.02036.x

[25] Wolf AE, Dietz KJ, Schroder P (1996) Degradation of glutathione S-conjugates by a carboxypeptidase in the plant vacuole. FEBS Lett 384: 31–34. 8797797 10.1016/0014-5793(96)00272-4

[26] Liu L, Wang J, Zhang Z, Zhao J, Pan G (2013) Cloning and Expression Analysis of Serine Carboxypeptidases in Maize (Zea mays L.). Acta Agronomica Sinica 39: 164–171.

[27] Zhang J, Guo D, Chang Y, You C, Li X, Dai X, et al (2007) Non-random distribution of T-DNA insertions at various levels of the genome hierarchy as revealed by analyzing 13 804 T-DNA flanking sequences from an enhancer-trap mutant library. Plant J 49: 947–959. 17253985 10.1111/j.1365-313X.2006.03001.x

[28] Xiao JH, Wu CY, Yuan M, Wang NL, Fang YR, Yang M, et al (2015) The progress and perspective of rice functional genomics research in China (in Chinese). Chin Sci Bull 60: 1711–1722.

[29] Washio K, Ishikawa K (1994) Organ-specific and hormone-dependent expression of genes for serine carboxypeptidases during development and following germination of rice grains. Plant Physiol 105: 1275–1280. 7972496 10.1104/pp.105.4.1275 PMC159459

[30] Liu H, Wang X, Zhang H, Yang Y, Ge X, Song F, et al (2008) A rice serine carboxypeptidase-like gene OsBISCPL1 is involved in regulation of defense responses against biotic and oxidative stress. Gene 420: 57–65. 10.1016/j.gene.2008.05.006 18571878

[31] Li Y, Fan C, Xing Y, Jiang Y, Luo L, Sun L, et al (2011) Natural variation in GS5 plays an important role in regulating grain size and yield in rice. Nat Genet 43: 1266–1269. 10.1038/ng.977 22019783

[32] Kawahara Y, de la Bastide M, Hamilton JP, Kanamori H, McCombie WR, Ouyang S, et al (2013) Improvement of the Oryza sativa Nipponbare reference genome using next generation sequence and optical map data. Rice (N Y) 6: 4. 24280374 10.1186/1939-8433-6-4 PMC5395016

[33] Lawrence CJ, Harper LC, Schaeffer ML, Sen TZ, Seigfried TE, Campbell DA, et al (2008) MaizeGDB: The maize model organism database for basic, translational, and applied research. Int J Plant Genomics 2008: 496957 10.1155/2008/496957 18769488 PMC2518694

[34] Wang L, Xie W, Chen Y, Tang W, Yang J, Ye R, et al (2010) A dynamic gene expression atlas covering the entire life cycle of rice. Plant J 61: 752–766. 10.1111/j.1365-313X.2009.04100.x 20003165

[35] Zhang S, Cai Z, Wang X (2009) The primary signaling outputs of brassinosteroids are regulated by abscisic acid signaling. Proc Natl Acad Sci U S A 106: 4543–4548. 10.1073/pnas.0900349106 19240210 PMC2657416

[36] Bai AN, Lu XD, Li DQ, Liu JX, Liu CM (2016) NF-YB1-regulated expression of sucrose transporters in aleurone facilitates sugar loading to rice endosperm. Cell Res 26: 384–388. 10.1038/cr.2015.116 26403192 PMC4783462

[37] Milla MA, Townsend J, Chang IF, Cushman JC (2006) The Arabidopsis AtDi19 gene family encodes a novel type of Cys2/His2 zinc-finger protein implicated in ABA-independent dehydration, high-salinity stress and light signaling pathways. Plant Mol Biol 61: 13–30. 16786289 10.1007/s11103-005-5798-7

[38] Li G, Tai FJ, Zheng Y, Luo J, Gong SY, Zhang ZT, et al (2010) Two cotton Cys2/His2-type zinc-finger proteins, GhDi19-1 and GhDi19-2, are involved in plant response to salt/drought stress and abscisic acid signaling. Plant Mol Biol 74: 437–452. 10.1007/s11103-010-9684-6 20852918

[39] Qin LX, Nie XY, Hu R, Li G, Xu WL, Li XB, et al (2016) Phosphorylation of serine residue modulates cotton Di19-1 and Di19-2 activities for responding to high salinity stress and abscisic acid signaling. Sci Rep 6: 20371 10.1038/srep20371 26829353 PMC4734338

[40] Wang L, Yu C, Chen C, He C, Zhu Y, Huang W, et al (2014) Identification of rice Di19 family reveals OsDi19-4 involved in drought resistance. Plant Cell Rep 33: 2047–2062. 10.1007/s00299-014-1679-3 25236158

[41] Xu C, Liu Y, Li Y, Xu X, Xu C, Li X, et al (2015) Differential expression of GS5 regulates grain size in rice. J Exp Bot 66: 2611–2623. 10.1093/jxb/erv058 25711711 PMC4986870

[42] Tabuchi M, Sugiyama K, Ishiyama K, Inoue E, Sato T, Takahashi H, et al (2005) Severe reduction in growth rate and grain filling of rice mutants lacking OsGS1;1, a cytosolic glutamine synthetase1;1. Plant J 42: 641–651. 15918879 10.1111/j.1365-313X.2005.02406.x

[43] Chastain CJ, Heck JW, Colquhoun TA, Voge DG, Gu XY (2006) Posttranslational regulation of pyruvate, orthophosphate dikinase in developing rice (Oryza sativa) seeds. Planta 224: 924–934. 16596412 10.1007/s00425-006-0259-3

[44] Asano T, Kunieda N, Omura Y, Ibe H, Kawasaki T, Takano M, et al (2002) Rice SPK, a calmodulin-like domain protein kinase, is required for storage product accumulation during seed development: phosphorylation of sucrose synthase is a possible factor. Plant Cell 14: 619–628. 11910009 10.1105/tpc.010454 PMC150584

[45] Ma B, He SJ, Duan KX, Yin CC, Chen H, Yang C, et al (2013) Identification of rice ethylene-response mutants and characterization of MHZ7/OsEIN2 in distinct ethylene response and yield trait regulation. Mol Plant 6: 1830–1848. 10.1093/mp/sst087 23718947

[46] Hobo T, Kowyama Y, Hattori T (1999) A bZIP factor, TRAB1, interacts with VP1 and mediates abscisic acid-induced transcription. Proc Natl Acad Sci U S A 96: 15348–15353. 10611387 10.1073/pnas.96.26.15348 PMC24822

[47] Zhang D, Chen L, Li D, Lv B, Chen Y, Chen J, et al (2014) OsRACK1 is involved in abscisic acid- and H2O2-mediated signaling to regulate seed germination in rice (Oryza sativa, L.). PLoS One 9: e97120 10.1371/journal.pone.0097120 24865690 PMC4035261

[48] Hrabak EM, Chan CW, Gribskov M, Harper JF, Choi JH, Halford N, et al (2003) The Arabidopsis CDPK-SnRK superfamily of protein kinases. Plant Physiol 132: 666–680. 12805596 10.1104/pp.102.011999 PMC167006

[49] Kato T, Sakurai N, Kuraishi S (1993) The changes of endogenous abscisic acid in developing grain of two rice cultivars with different grain size. Japanese journal of crop science 62: 456–461.

[50] Zhang H, Tan G, Yang L, Yang J, Zhang J, Zhao B, et al (2009) Hormones in the grains and roots in relation to post-anthesis development of inferior and superior spikelets in japonica/indica hybrid rice. Plant Physiol Biochem 47: 195–204. 10.1016/j.plaphy.2008.11.012 19117763

[51] Ahmadi A, Baker DA (1999) Effects of abscisic acid (ABA) on grain filling processes in wheat. Plant Growth Regulation 28: 187–197.

[52] Shu K, Liu XD, Xie Q, He ZH (2016) Two Faces of One Seed: Hormonal Regulation of Dormancy and Germination. Mol Plant 9: 34–45. 10.1016/j.molp.2015.08.010 26343970

[53] Nakashima K, Yamaguchi-Shinozaki K (2013) ABA signaling in stress-response and seed development. Plant Cell Rep 32: 959–970. 10.1007/s00299-013-1418-1 23535869

[54] Hoecker U, Vasil IK, McCarty DR (1995) Integrated control of seed maturation and germination programs by activator and repressor functions of Viviparous-1 of maize. Genes Dev 9: 2459–2469. 7590227 10.1101/gad.9.20.2459

[55] McKibbin RS, Wilkinson MD, Bailey PC, Flintham JE, Andrew LM, Lazzeri PA, et al (2002) Transcripts of Vp-1 homeologues are misspliced in modern wheat and ancestral species. Proc Natl Acad Sci U S A 99: 10203–10208. 12119408 10.1073/pnas.152318599 PMC126648

[56] Fan J, Niu X, Wang Y, Ren G, Zhuo T, Yang Y, et al (2007) Short, direct repeats (SDRs)-mediated post-transcriptional processing of a transcription factor gene OsVP1 in rice (Oryza sativa). J Exp Bot 58: 3811–3817. 18057047 10.1093/jxb/erm231

[57] Giraudat J, Hauge BM, Valon C, Smalle J, Parcy F, Goodman HM, et al (1992) Isolation of the Arabidopsis ABI3 gene by positional cloning. Plant Cell 4: 1251–1261. 1359917 10.1105/tpc.4.10.1251 PMC160212

[58] Nambara E, Naito S, Mccourt P (1992) A mutant of Arabidopsis which is defective in seed development and storage protein accumulations is a new abi3 allele. Plant Journal 2: 435–441.

[59] Zhang J, Nallamilli BR, Mujahid H, Peng Z (2010) OsMADS6 plays an essential role in endosperm nutrient accumulation and is subject to epigenetic regulation in rice (Oryza sativa). Plant J 64: 604–617. 10.1111/j.1365-313X.2010.04354.x 20822505

[60] Miki D, Shimamoto K (2004) Simple RNAi vectors for stable and transient suppression of gene function in rice. Plant Cell Physiol 45: 490–495. 15111724 10.1093/pcp/pch048

[61] Hou Y, Wang L, Wang L, Liu L, Li L, Sun L, et al (2015) JMJ704 positively regulates rice defense response against Xanthomonas oryzae pv. oryzae infection via reducing H3K4me2/3 associated with negative disease resistance regulators. BMC Plant Biol 15: 286 10.1186/s12870-015-0674-3 26646110 PMC4673860

[62] Livak KJ, Schmittgen TD (2001) Analysis of relative gene expression data using real-time quantitative PCR and the 2(-Delta Delta C(T)) Method. Methods 25: 402–408. 11846609 10.1006/meth.2001.1262

