# Supplementary figures and images for: Serine carboxypeptidase 46 Regulates Grain Filling and Seed Germination in Rice (Oryza sativa L.)
Source: PLoS One. 2016 Jul 22;11(7):e0159737. doi: 10.1371/journal.pone.0159737 (PMC4957776; doi:10.1371/journal.pone.0159737)

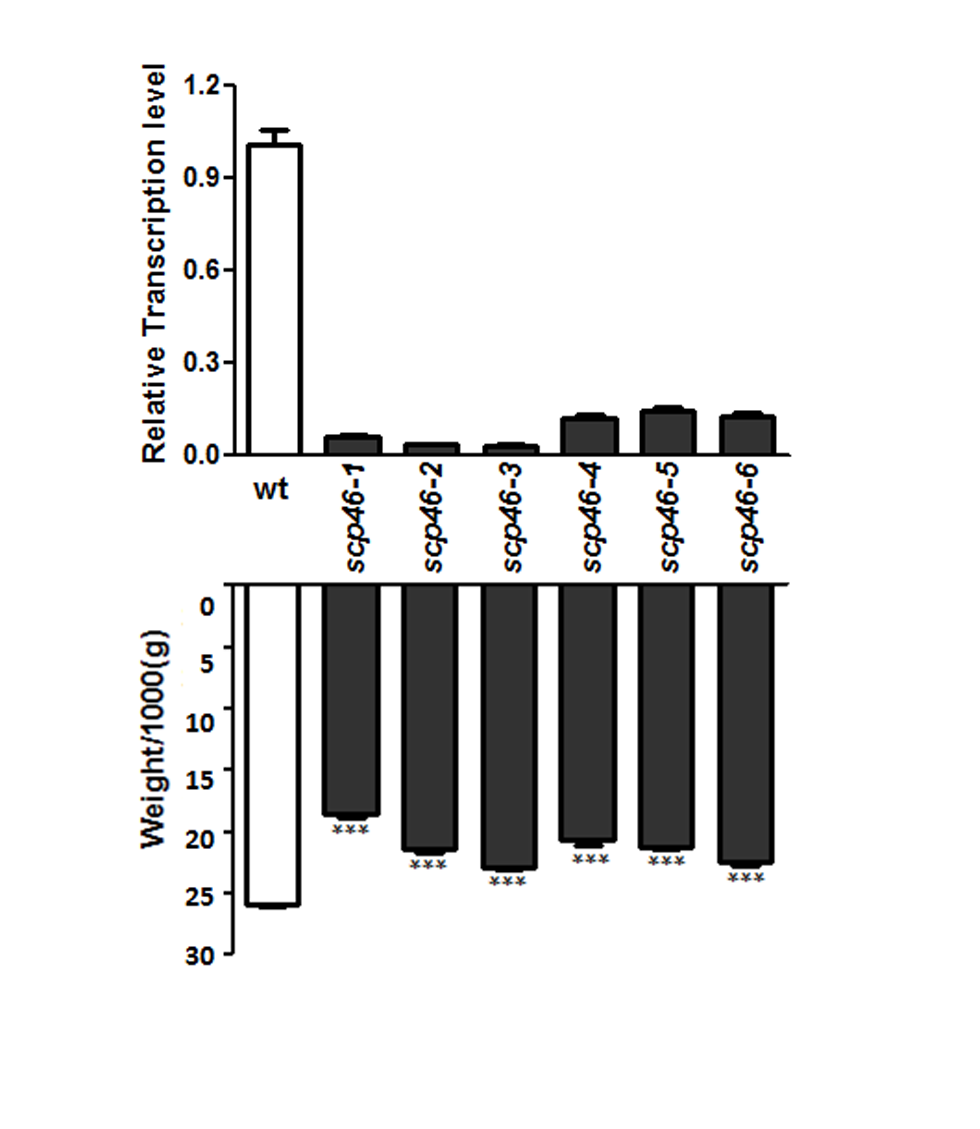

Supplement: S1 Fig — (TIF) [file pone.0159737.s001.tif]

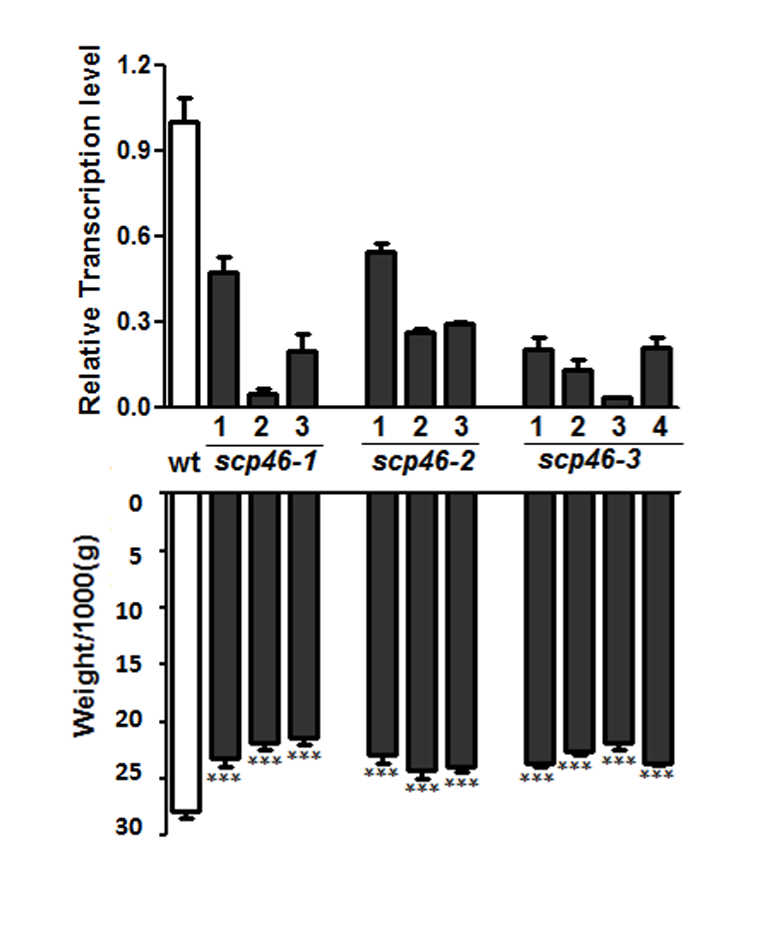

Supplement: S2 Fig — (TIF) [file pone.0159737.s002.tif]

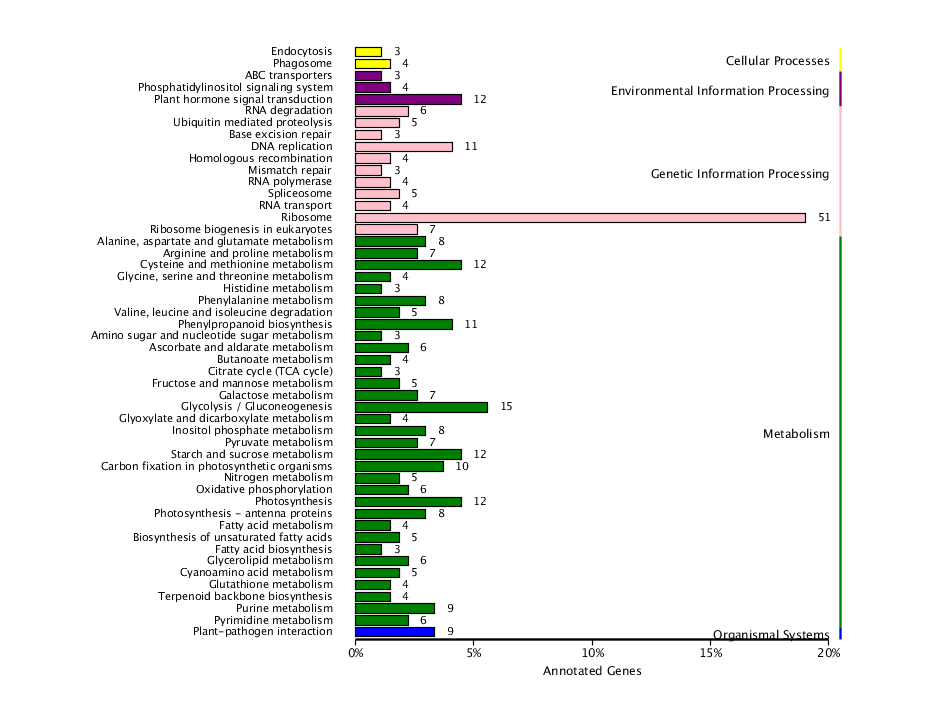

Supplement: S3 Fig — (TIF) [file pone.0159737.s003.tif]
